# Efficiency in the governance of the Covid-19 pandemic: political and territorial factors

**DOI:** 10.1186/s12992-021-00759-4

**Published:** 2021-09-21

**Authors:** Pedro-José Martínez-Córdoba, Bernardino Benito, Isabel-María García-Sánchez

**Affiliations:** 1 Department of Accounting and Finance, Faculty of Economics and Business, Regional Campus of International Excellence “Campus Mare Nostrum”, University of Murcia, 30100 Murcia, Spain; 2 Instituto Multidisciplinar de Empresa, Campus Miguel de Unamuno, Universidad de Salamanca, 37007 Salamanca, Spain

**Keywords:** Covid-19, Efficiency, Pandemic governance, Global crisis, Economic impacts, Undesirable factors

## Abstract

**Background:**

The pandemic generated by Covid-19 has changed the way of life of citizens around the world in a short time, affecting all areas of society directly or indirectly, which is facing a global health crisis with different national responses implemented by governments. Several months into the pandemic, the first after-effects of Covid-19 are beginning to be felt by citizens, who are questioning the management carried out so far. In order to improve the performance of governmental decisions to reduce the impact of the pandemic during the coming months, we calculated the levels of efficiency in the management of health resources. In addition, we identify some country characteristics that may condition efficient management.

**Results:**

We obtained significant differences according to the geographical location of the country, with European and American countries being less efficient than Asian and African countries. Likewise, we can affirm that greater freedom of expression, a higher median age and an unstable economy and labor market reduce efficiency. However, female leadership of the government and greater compliance with the rule of law offer more efficient management, as do countries that derive more revenues from tourism.

**Conclusions:**

These results provide an opportunity for political leaders to reflect on their management during these months of the pandemic in order to identify mistakes and improve the implementation of effective measures. It has been shown that using more resources does not mean managing better; therefore, policymakers need to pay special attention to the use of resources, taking into account the budgetary constraints of the public sector.

## Introduction

On March 11, 2020, the World Health Organization (WHO) declared the disease caused by the coronavirus SARS-CoV-2, better known as Covid-19, to be a pandemic. Since then, several months later and with more than 100 million people infected and 2,2 million dead (by early February 2021), the world and its inhabitants have experienced events unusual for their time. With the fear of a new economic recession and its implications, in addition to the foreseeable waves of infected people, an efficient management is required, capable of dealing with everything that exists and is about to arrive.

The characteristics of the Covid-19 pandemic, due to its severity, immediacy and complexity, have highlighted the weaknesses of governments in solving this crisis. Zoonotic diseases (such as that caused by Covid-19) represent a threat to life in society, with the possibility of causing a serious disruption to the world economy, a global event whose response is national and depends on the behavior patterns of citizens.

The socio-economic effects of Covid-19 are many and diverse, individually and collectively damaging people and the economy, and appropriate management is needed to resolve this situation [34]. Covid-19 is a challenge in terms of public health that affects all areas of life, where public managers responsible for solving this crisis must manage carefully and proactively the available resources in order to avoid risks and reduce the impact of the pandemic.

Effective management, able to take decisive action based on scientific knowledge rather than political opportunity, can explain much of the success of the response to Covid-19. Coordination, resource availability and political accountability can contribute to this goal, although many of the decisions adopted during the current pandemic have focused on political and economic considerations, and have left aside public health aspects [20].

In this sense, it is difficult to understand the different responses that governments have adopted to the same situation. Indeed, questions related to the governance of the pandemic arise, such as: Which territories have better managed health resources? Does political ideology influence the management of the pandemic? Has the governance of the pandemic been efficient? Thus, we propose three dimensions - Territory, Politics and Governance - interrelated with the efficient management of the pandemic [16], which may explain the differences between countries.

This research aims to understand the efficiency in the management of health resources to cope with the pandemic. Furthermore, we identify the impact of the country’s characteristics (territorial, political, governance, sociodemographic and economic) that can condition the transmission of the virus, and consequently efficient management. Taking into account the volatility of the pandemic, the availability of information and the methodological changes that make it difficult to obtain data, we conducted an analysis that allows us to obtain the levels of efficiency for 155 countries along with results capable of contributing to improving the management of the pandemic over the coming months and years.

## Theoretical framework

The need to find solutions to an unusual situation that affects all aspects of life has served to unite academia around Covid-19. In fact, as of January 1, 2021, in the main collection of the Web of Science we located 63,708 results when searching for the term “Covid-19”, among which 32,782 are articles, which shows the relevance of the problem in just a few months. We reviewed some publications related to the management of Covid-19 and its impact on social, economic, and political areas. Below we highlight the most relevant aspects related to policy, pandemic governance and territory, as well as research related to efficiency in health management.

### Literature in times of pandemic

The Covid-19 pandemic has shown how a health crisis can cause unprecedented damage worldwide. Other tragic events such as climate change or localized catastrophes allow for a greater response capacity, while the dimensions of this pandemic are yet to be discovered [21]. Some effects are already visible with the paralysis of economic activity, which represents a serious risk to the general and socioeconomic well-being of people [33].

The trade-off between economics and health has led to an important debate on how to take the most effective measures to curb the impact of the pandemic. The intensity and speed of the economic shock, highly visible in the loss of employment, and the severity of the economic contraction in relation to the spread of the virus have been the first consequences. All this has led to economic uncertainty never before seen on a global scale, which will make a rapid and complete recovery difficult [2].

Thus, differences in economic forecasts are related to the response capacity implemented by governments, as well as their exposure to international transmission of the virus, especially those most dependent on tourism. It is expected that governments with better performance in managing the crisis will also do so in economic terms, i.e., good health management of the pandemic is profitable for the country’s society and economy [30].

In this regard, the speed and nature of post-Covid-19 economic recovery will be hampered by government actions to curb the pandemic, such as numerous disruptions in supply chains as a result of confinement and the reduction in demand due to decreased activity to avoid contracting the disease [44]. Consequently, it is necessary to diversify economic activities and reduce dependence on specific sectors to mitigate the impact of similar situations that may occur in the near future; in addition, investment in health infrastructure to deal with infectious diseases and progress in information and communication technologies is needed if pre-pandemic levels of growth and well-being are to be achieved [35].

The measures implemented to halt the spread of the virus have not had the same result in all countries [5], nor even within the same territory. The economic conditions of the population to cope with the health measures represent a relevant difference. Thus, citizens with inadequate housing, high rates of poverty or unemployment have a higher risk of death from Covid-19, even among the youngest population [22]. In the case of Italy, the first Western country to be severely punished by the pandemic, the reduction of mobility has been transcendental in the impact of the pandemic, with citizens with higher economic levels being more compliant, as they do not have to leave their homes to obtain resources [6].

### Political

In addition, public leadership, not only political but also health-related, is presented as a key element in improving the management of the pandemic [19]. Leaders must be institutionally prepared for change and open to public-private collaboration to improve health management. These changes must be coupled with greater transparency in public health decisions, ensuring that science is not overridden by ideology, even when politically motivated [38]. In this sense, countries led by women tend to listen to and trust more the recommendations of science. Traditionally female characteristics, such as empathy, compassion and caring, have led to more effective responses to Covid-19 [37], improving governance in times of the pandemic.

In democratic systems, government legitimacy is an indispensable condition for maintaining political capacity and credibility. In times of crisis, citizens rely on government for credible information to guide their individual behavior [25]. Although ideological differences during the pandemic months have not had a significant impact on policy decisions, an early response has great advantages over a strict delay, i.e., early and flexible actions to contain the virus have better results than late and severe ones [40].

Notwithstanding the above, in some prominent countries the implementation of measures has been conditioned by the ideology of the rulers. For example, in the United States, Republicans are not as keen on following orders of social distancing as Democrats, the latter being more inclined to maintain recommended distances for the population and to comply with mobility restrictions [36]. In the case of Spain, and after the first wave of contagion, citizens are directing their preferences towards technocratic governments with strong leadership, given that the diversity of political opinions has not translated into the containment of the pandemic [3].

### Governance of pandemics

The public sector faces complex problems in an increasingly turbulent social environment, having to manage uncertain and unpredictable scenarios. At the same time, it tries to solve these problems under pressure and without sufficient knowledge of their cause and effect. This requires political leaders to improve their responsiveness by designing, combining and executing sound governance strategies [4].

One of these problems has to do with pandemic diseases, which are capable of undermining even the best pre-established plans, due to their unprecedented characteristics and divergent requirements for their solution, irrespective of each government’s forecasts. In this regard, governments face several important constraints in the governance of the pandemic, in particular with the uncertainty of citizens about the adverse consequences of the pandemic [10].

In this sense, the challenges posed by the governance of pandemics are not simply technical, but adequate government management must also take into account socio-political issues as well as the media projection of the events. A rational scientific approach to the management of pandemics is insufficient in the current socio-demographic and globalised context, and a socio-political mix of science, culture and public perceptions is needed for the development of public health policies [7]. Effective pandemic management requires an adaptive (taking into account the unprecedented character of the events) learning approach by governments, as well as a combination of knowledge of public health, epidemiology and socio-political factors, where trust in institutions, leadership or governance can be key elements [24].

Thus, the exercise of adequate governance can determine the outcomes of pandemic disease management. In the case of Human Immunodeficiency Virus (HIV), it has been shown that inadequate governance is associated with a higher prevalence of the disease, while it has been found that as governance improves, fewer women die in childbirth, there are more doctors per inhabitant, there is better access to clean water and life expectancy increases [32]. Furthermore, citizens’ trust in government, as well as compliance with imposed rules and acceptance of new norms and values, is fundamental to the implementation of valid pandemic governance solutions, since government recommendations will be subject to frequent reformulations that may test the population’s understanding and comprehension [4].

Predictive pandemic governance models provide robust and reliable evidence for decision-making. It is easy to think, therefore, that such models yield firm and reliable evidence, although during the swine flu pandemic in 2009, the weakness of the evidence formulated in the prediction was noted. Indeed, as time passed and cases were reported, the following questions were asked: Was a pandemic alert necessary? Why did governments spend a significant amount of money on vaccines and antiviral stockpiles that were never used and have now expired? [31]. Nevertheless, predictive models must be seen as a form of technical rationality in the broader context of governance [14]. Hence the timeliness of our study, in the sense that the results obtained can help governments in their investment decisions in the face of possible health pandemics that are likely to occur in the future.

Another important aspect of improving the speed and efficiency of pandemic governance is to learn from the past and adapt institutions to the new reality. A good example of this is the case of South Korea in the management of Middle East Respiratory Syndrome (MERS) in 2015, where after overcoming the pandemic they implemented new policies and institutional changes in anticipation of future pandemics, which has favoured the governance of the new coronavirus (Covid-19) with the early introduction of effective measures [38].

### Territory

The Covid-19 pandemic became relevant in China in early 2020, and in March 2020 the world was confined without knowing how to deal with an unprecedented situation that was spreading out of control. It has been shown that the risk posed by lax health regulation in one locality can easily and quickly lead to a global health crisis (as has happened), and it is necessary to identify the geographical areas where the convergence of risk factors is most intense [9].

On the other hand, the supervening difficulties caused by a pandemic make it necessary to reflect on the appropriateness of whether public health governance and decision-making should be elevated to the global level. Subsidiarity provides a means in this regard to consider whether these public powers should be reallocated, even temporarily, although public health and economics are fundamental values within each State [15]. Subsidiarity is understood as the appropriate geographical distribution of power, arguing that powers should rest at the lowest possible level, unless it is more effective to allocate them at a higher level.

### Efficient health management

The limited economic resources of the public sector, together with the citizens’ demands for quality health care, force politicians to innovate in management to be more efficient. From this point of view, traditional management has been compared to *New Public Management*, without finding significant differences. However, numerous studies have shown greater efficiency in public health management than in private management [27]. For example, this is the case in Spain [1] or Germany, where privately owned healthcare centers show lower levels of efficiency, explained in part by a longer stay in these centers than in the public ones [26].

On the other hand, Hafidz et al. [23] suggest a series of recommendations to politicians with the aim of improving health services on both the supply and demand sides. On the supply side, it would be appropriate to optimize the workforce and the infrastructure, increase the quality of service and develop financial strategies; while on the demand side, financial barriers should be minimized, accessibility to health services should be increased and citizens’ health habits should be changed. In addition, a system of hospital costs that allows an exhaustive control would mean taking more efficient decisions, thus improving the performance in health management [18]. However, in order to analyze health management in terms of efficiency, it is necessary to include exogenous factors that can strongly condition the provision of the service [11].

## Methods

### Sample

**Fig. 1:**
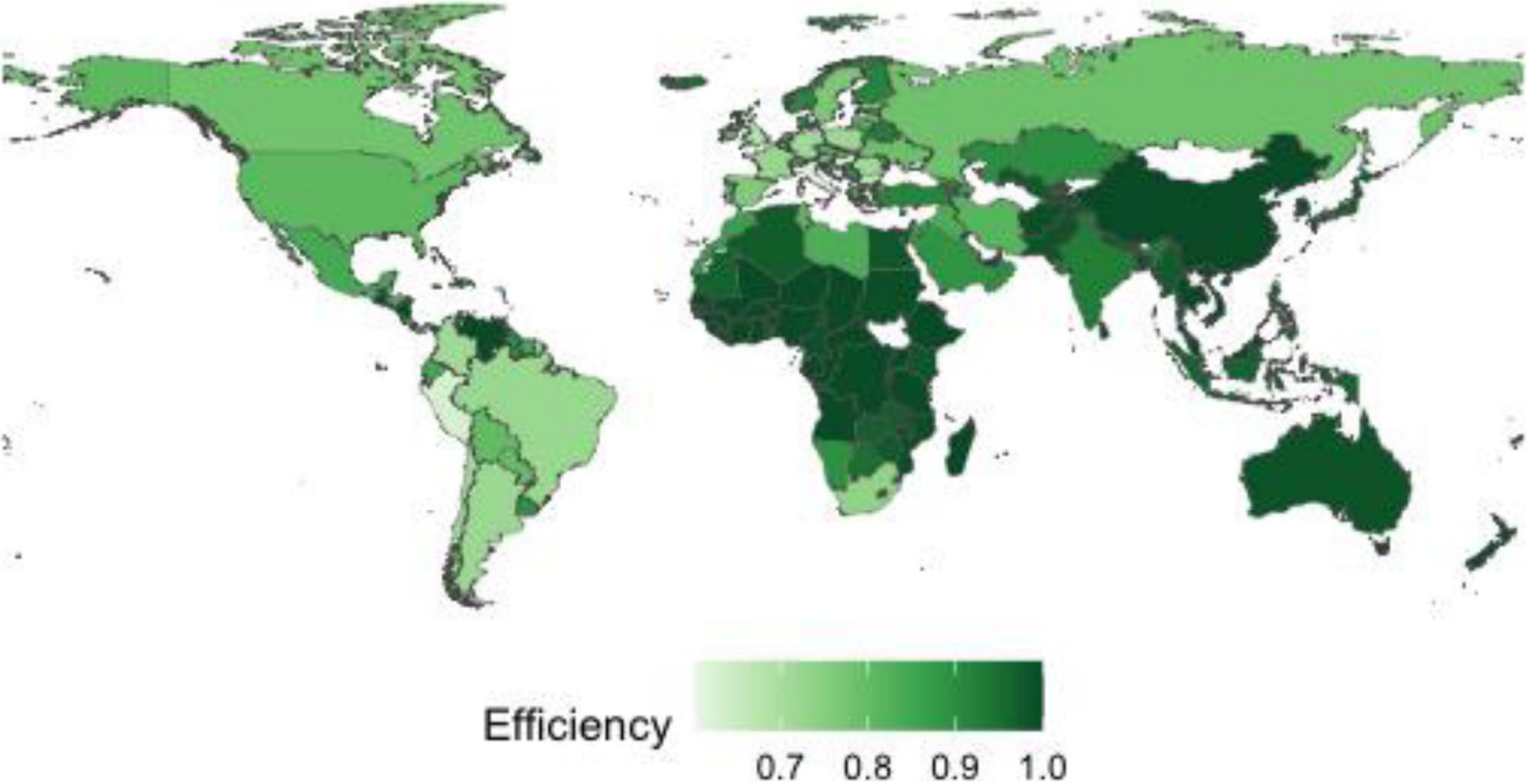
Graphic representation of efficiency levels

**Fig. 2:**
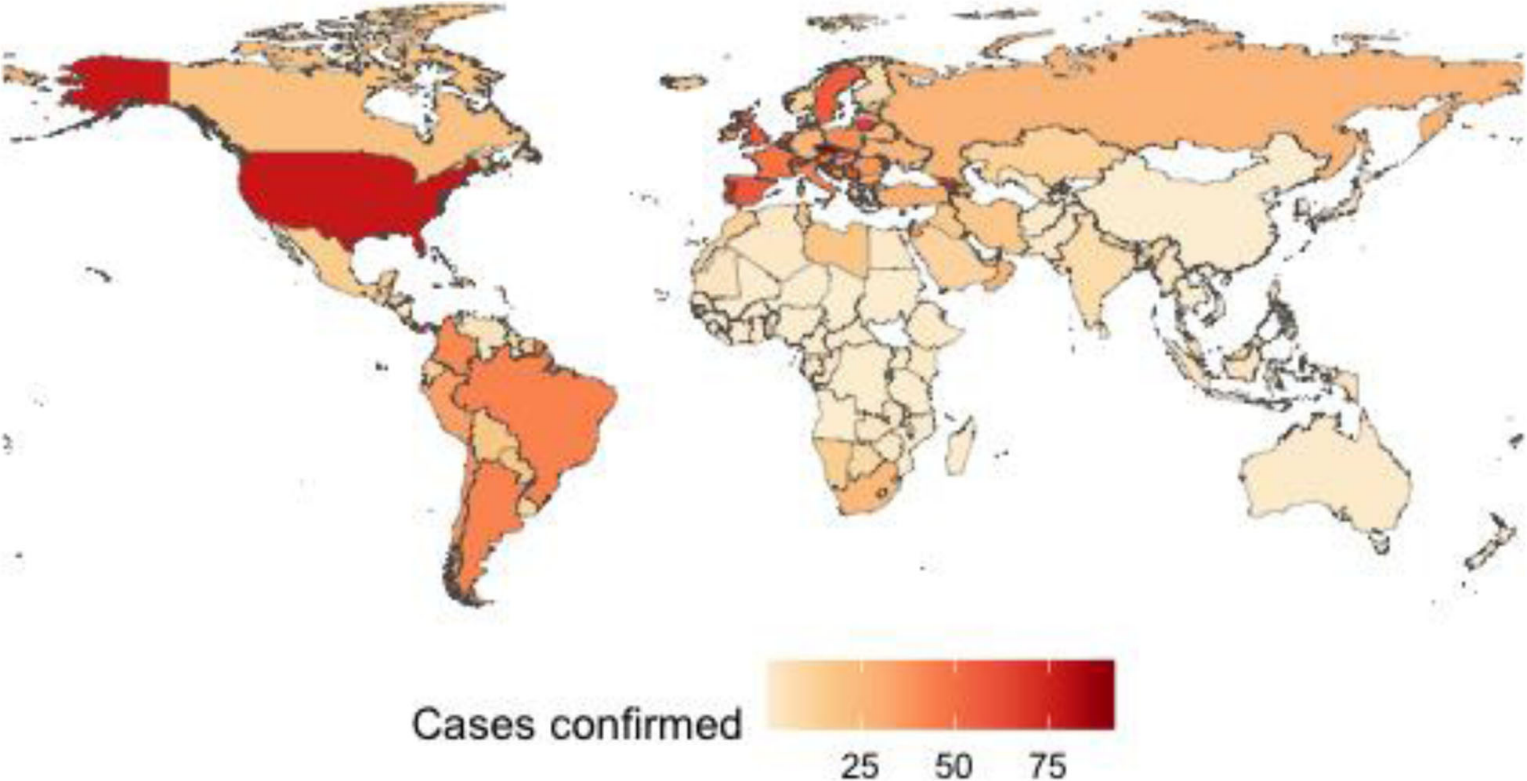
Graphic representation of the output *Cases confirmed*

The speed and volatility of the pandemic has meant a complicated statistical process to homogenize data and provide valid and reliable information is needed. In order to analyze the level of efficiency in the management of the pandemic, it is necessary to have a certain number of comparable observations. The data offered by international organizations (World Bank, WHO, International Monetary Fund (IMF)) regarding the 237 countries of the world, are considered the most appropriate sources of information today. In addition, since the beginning of the pandemic, management has been led by national governments, which set the guidelines and standardize decisions at the country level. Therefore, the most appropriate units of analysis are the countries, although we have had to select those that report on the variables needed to calculate efficiency (see Table 1). Thus, once the countries with incomplete or erroneous data have been filtered out, the final sample was a set of 155 countries. Figures 1 and 2 show the countries included in the sample, with the exclusion of a small group that did not adequately report the necessary variables.

**Table 1:** Description and descriptive statistics of the inputs, outputs, efficiency and environmental variables

Variable	Description	Min	Mean	Median	Max	Standard deviation
*Inputs*						
Physicians	Total number of physicians per thousand inhabitants^a^	0.0140	1.9042	1.6090	7.1201	1.6125
Nurses	Total number of nurses, midwives and other associated personnel per thousand inhabitants^a^	0.0737	4.5672	2.9460	19.4614	4.3775
Hospital beds	Beds available in public and private hospitals per thousand inhabitants^a^	0.1000	2.8419	2.2000	13.4000	2.3871
Health expenditure	Level of current health expenditure expressed as a percentage of GDP^a^	1.1812	6.5678	6.4294	17.0613	2.5149
*Outputs*						
Cases confirmed	Total number of confirmed cases of Covid-19 per thousand inhabitants as February 1, 2021^b^	0.0088	20.5396	11.7416	92.5357	22.7485
Death rate	Total number of deaths with Covid-19 per thousand confirmed cases as February 1, 2021^b^	0.4871	20.5774	17.7672	85.1128	12.9607
*Dependent variable*						
Efficiency	Own elaboration from DEA technique	0.6018	0.8965	0.9399	1.0000	0.1092
*Environmental variables*						
Europe	Dummy variable that takes the value 1 if the country belongs to the European region established by the WHO and 0 otherwise^b^	0.0000	0.2968	0.0000	1.0000	0.4583
Americas	Dummy variable that takes the value 1 if the country belongs to the American region established by the WHO and 0 otherwise^b^	0.0000	0.1871	0.0000	1.0000	0.3913
Asia	Dummy variable that takes the value 1 if the country belongs to the regions of South-East Asia and Western Pacific established by the WHO and 0 otherwise^b^	0.0000	0.1226	0.0000	1.0000	0.3290
Africa	Dummy variable that takes the value 1 if the country belongs to the regions of Africa and Eastern Mediterranean established by the WHO and 0 otherwise^b^	0.0000	0.3935	0.0000	1.0000	0.4901
Gender	Dummy variable that takes the value 1 if the government leader is a woman and 0 if it is a man^c^	0.0000	0.1032	0.0000	1.0000	0.3052
Ideology	Dummy variable that takes the value 1 if the government leader’s ideology is conservative and 0 if it is progressive^c^	0.0000	0.4839	0.0000	1.0000	0.5014
Voice	Quality of freedom of expression, association, media, and citizen participation in the election of government, which takes values between 0 and 100^d^	2.4631	48.5238	45.3202	100.0000	28.4050
Rule of law	Society’s compliance with the rule of law, which takes values between 0 and 100^d^	0.4808	48.3468	46.1538	100.0000	28.6079
Median age	Median age of the population^f^	15.1510	30.5919	30.2620	48.3580	9.3129
GDP	Forecast year-on-year change in gross domestic product at constant prices as a percentage for 2019^e^	−35.0000	2.5109	2.3350	9.8900	4.1384
Tourism	Income from international tourism as a percentage of total exports in goods and services^a^	0.1888	13.7942	7.7834	85.2048	16.4688
Unemployment	Unemployment rate forecast for 2019^e^	0.2950	7.1966	7.1966	28.7000	3.7324

^a^Data from https://data.worldbank.org/indicator?tab=all Accessed February, 2021

^b^Data from https://covid19.who.int/table Accessed February, 2021

^c^Own elaboration after consulting web information about the country

^d^Data from http://info.worldbank.org/governance/wgi/ Accessed February, 2021

^e^Data from https://www.imf.org/en/Publications/WEO/weo-database/2020/October Accessed February, 2021

^f^Data from https://population.un.org/wpp/Download/Standard/Population/ Accessed July, 2021

### Efficiency

The research context determines the most appropriate technique for measuring efficiency. In our case, the characteristics of the public sector make it necessary to select a method that easily manages the production function and does not require data on the price of inputs or outputs, which is difficult to obtain in public services [41]. Among the most commonly used techniques, non-parametric (Data Envelopment Analysis -DEA, Free Disposable Hull and Order-m) and parametric (Stochastic Frontier Analysis) methods are the most used, with DEA being the most appropriate for calculating efficiency in the public sector environment, and more specifically in health care [23, 28, 39]. The objective of DEA is to obtain a relative efficiency level by means of linear programming problems, forming a frontier (envelope) that incorporates all the efficient Decision-Making Units (DMUs) (best input-output ratio) and their linear combinations, while placing the rest of the DMUs with values lower than the unit as inefficient.

To calculate the levels of efficiency with DEA, the inputs and outputs must be selected, and these are will be determined by the research objective. In our case, to determine the efficiency of pandemic management, the inputs refer to the resources available to manage a health crisis of these characteristics, and the outputs to its direct consequences. Thus, following the most recent literature analyzing efficiency in the health sector [17, 29], the selected inputs are the available physicians (*Physicians*) and nurses (*Nurses*), the number of hospital beds (*Hospital beds*) and the current expenditure on health care (*Health expenditure*), while the number of people infected by Covid-19 (*Cases confirmed*) and the number of deaths (*Death rate*) form the outputs. Table 1 explains each input and output in more detail, as well as the sources of information and descriptive statistics.

With the *R-Studio* software and the *deaR* package [8] we have obtained the efficiency levels for each country. DEA allows us to select the orientation of the model between input, when the objective is to minimize the resources employed (inputs) while maintaining constant the results (outputs), or output, when the aim is to maximize the results (outputs) while maintaining the resources (inputs). In our case, it could be interesting to minimize the vector of inputs (*Physicians*, *Nurses*, *Hospital beds* and *Health expenditure*) or to maximize the vector of outputs (*Cases confirmed* and *Death rate*). However, what we intend in our research is to design a model that allows us to maintain or increase the vector of inputs as far as possible and incorporate a vector of undesired outputs. Thus, we opt for the output orientation according to the model proposed by Seiford and Zhu [42], which reduces the undesired outputs. In addition, we selected variable returns to scale (VRS) due to the differences in the size of the DMUs, where different scales of production can be developed.

### Environmental variables

Following the literature reviewed in the previous section (Theoretical framework), and in order to understand the characteristics of the countries that can influence the efficient management of the Covid-19 pandemic, we have selected variables representative of territory (*Europe*, *America*, *Asia* and *Africa*), politics (*Gender* and *Ideology*) and governance (*Voice* and *Rule of law*) of each country, as well as a set of control variables related to demographics (*Median age*) and economics (*GDP, Tourism* and *Unemployment*).

The variables *Europe, Americas, Asia* and *Africa* reflect the health regions established by the WHO. *Gender* represents the gender of the national government leader, which takes the value 1 if female and 0 for male. *Ideology* takes the value 1 for conservative rulers and 0 for progressive ones. Among the governance indicators we have selected those directly related to the management of the Covid-19 pandemic (*Voice* and *Rule of law*). *Voice* indicates freedom of expression, freedom of association and freedom of the media, and by choosing it we intend to show governments’ commitment to transparency. *Rule of law* reflects compliance and respect for established rules. *Median age* represents the median age of the population. *GDP* is the percentage of year-on-year change in constant prices of Gross Domestic Product for 2019. *Tourism* is the percentage of exports in goods and services that represent the income obtained from international visitors. *Unemployment* is the unemployment rate in 2019. Table 1 specifies in more detail these variables, their descriptive statistics and the sources of information.

### Regression model

To determine the impact of environmental variables on efficiency levels, we estimated the following regression model:

1 $$ {\hat{\delta}}_i=\alpha +{\beta}_1 Europ{e}_i+{\beta}_2 Americ\mathrm{a}{s}_i+{\beta}_3 Asi{a}_i+{\beta}_4 Afric{a}_i+{\beta}_5 Gende{r}_i+{\beta}_6 Ideolog{y}_i+{\beta}_7 Voic{e}_i+{\beta}_8 Rule\ of\  la{w}_i+{\beta}_9 Median\  ag{e}_i+{\beta}_{10} GD{P}_i+{\beta}_{11} Touris{m}_i+{\beta}_{12} Unemploymen{t}_i+{\varepsilon}_i $$

where ${\hat{\delta}}_i$ represents the level of efficiency for each country; *α* is the constant of the model; *β*_*j*_ are the coefficients of each variable; *Europe*, *Americas*, *Asia*, *Africa*, *Gender*, *Ideology*, *Voice*, *Rule of law*, *Median age*, *GDP*, *Tourism* and *Unemployment*, are the environmental variables; and *ε*_*i*_ is the term of error.

The level of efficiency obtained with DEA, defined in the interval [0–1], conditions us to a truncated regression model as the best option to test the impact of environmental variables in a second stage [43]. We used *R-Studio* software with the package *truncreg*, which estimates model (1) for truncated Gaussian variables by maximum likelihood [12]. In addition, to avoid possible biases in the efficiency calculation, the separability condition [13] between inputs-outputs and environmental variables was tested, and the independence of these was confirmed.

The correlation matrix between inputs-outputs and environmental variables (Table 2) shows the significance of some of these variables, without compromising the validity of the level of efficiency or the regression model (1). For example, the indissoluble link between *Physicians* and *Nurses*, who complement each other in the healthcare activity, is essential to respond to the pandemic. In the case of the environmental variables, the significance is centered on aspects related to the geographical situation of the country (*Europe*, *Americas*, *Asia* and *Africa*), which we regress alternatively to avoid perfect multicollinearity.

**Table 2:** Coefficient of correlation among inputs-outputs and environmental variables

***Inputs-Outputs***											
	**Physicians**	**Nurses**	**Hospital beds**	**Health expenditure**	**Cases confirmed**						
Nurses	***0.6773	1.0000									
Hospital beds	***0.6526	***0.5949	1.0000								
Health expenditure	***0.4247	***0.5122	***0.3134	1.0000							
Cases confirmed	***0.6539	***0.5337	***0.3989	***0.4089	1.0000						
Death rate	−0.1118	−0.1779	− 0.1035	0.1340	− 0.1005						
***Environmental variables***											
	**Europe**	**Americas**	**Asia**	**Africa**	**Gender**	**Ideology**	**Voice**	**Rule of law**	**Median age**	**GDP**	**Tourism**
Americas	**-0.3117	1.0000									
Asia	−0.2428	−0.1793	1.0000								
Africa	***-0.5233	***-0.3865	**-0.3011	1.0000							
Gender	0.2438	−0.0540	0.0672	−0.2299	1.0000						
Ideology	0.2470	−0.0011	−0.1257	− 0.1458	0.0110	1.0000					
Voice	***0.4351	0.1725	−0.0382	***-0.5190	**0.2962	0.2188	1.0000				
Rule of law	***0.4641	−0.0999	0.1354	***-0.4451	**0.3003	0.2596	***0.7771	1.0000			
Median age	***0.6808	0.0007	0.1257	***-0.7216	*0.2652	0.2350	***0.6245	***0.7520	1.0000		
GDP	0.0647	*-0.2939	0.1290	0.0876	0.0474	−0.0061	−0.0009	0.0485	−0.1038	1.0000	
Tourism	−0.1434	0.1676	0.0550	−0.0366	0.0212	−0.0033	0.0907	0.0142	−0.0665	0.0710	1.0000
Unemployment	−0.0719	0.0246	−0.2461	0.2128	−0.1427	−0.0858	− 0.0813	−0.1811	− 0.1416	−0.0652	0.1823

Significance: ***1%, **5%, *10%

Moreover, there may be correlations between the governance variables chosen, given that in countries with a strong *Rule of law*, the freedoms of expression, participation and communication (*Voice*) will be respected to a greater extent. In any case, taking into account the results of the control variables (see Table 4), the significant correlations shown in Table 2 do not condition the regression model (1).

## Discussion

The efficiency levels are shown in Fig. 1, where we observe a significant difference between regions (*Europe, Americas, Asia* and *Africa*). The countries of Europe and Americas obtain on average lower values compared to those of *Asia* and *Africa* (see Table 3). We found that the less efficient countries, which are represented with a lighter color in Fig. 1, obtain a darker color in Fig. 2. Thus, the countries of *Europe* and the *Americas* show a higher average incidence (*Cases confirmed*) than the countries of *Asia* and *Africa* (see Table 3).

**Table 3:** Average value of inputs, outputs and efficiency by geographical region

	*Europe*	*Americas*	*Asia*	*Africa*
Physicians	3.6686	1.9782	1.6531	0.6167
Nurses	8.7961	3.4903	4.8689	1.7961
Hospital beds	5.1000	2.0552	3.2895	1.3738
Health expenditure	7.9148	7.2415	5.3588	5.6083
Cases confirmed	42.4300	22.2633	4.6839	8.1513
Death rate	18.6998	26.4294	16.8990	20.3571
Efficiency	0.8041	0.8481	0.9833	0.9622

If the countries of Europe and the Americas have more resources (inputs) for pandemic management (see Table 3), their results should be more favorable. If this is not the case, we confirm the inefficiency in the management of health resources by these countries. These data could be explained by the capacity and information acquired in recent decades in Asian countries, as a result of having effectively combated similar viruses (SARS and MERS-CoV). This is also the case in African countries, where they coexist with more uncontrolled diseases (Ebola and Malaria) that bestow on society a greater awareness of the extraordinary measures of health protection. Although the average efficiency of Africa is higher than that of Europe or Americas, some countries with economic and tourism solvency are below the average of the latter, as is the case of South Africa or Tunisia (0.7352 and 0.8093, respectively).

During the first weeks of the pandemic, political leaders in some countries such as the United States, Brazil and the United Kingdom denied the extent and consequences of the virus, implementing measures that favoured its spread. Thus, the efficiency levels obtained in these countries (0.8084; 0.7215; 0.6809, respectively) do not correspond to their economic, political and social characteristics. On the opposite side we find the Asian country where the virus originated (China), or those with more experience in pandemic management for having solved similar situations (South Korea), with very high levels of efficiency (0.9993; 0.9841, respectively). On the other hand, the arrival of Covid-19 in European countries such as Germany, France and Spain tested the response capacity of their governments, which tried to improve on the management carried out by the first western country affected by the pandemic (Italy); however, the results confirm that more and better can be done (0.7153; 0.7248; 0.7567; 0.6018, respectively).

The estimation of the regression model (1) provides information on the effect of environmental variables on efficiency levels (see Table 4). Taking into account the perfect multicollinearity that would exist if we regressed the variables *Europe*, *Americas*, *Asia* and *Africa* in the same model, we performed four regressions, alternatively omitting one of these variables and maintaining the rest (Full model), to secure more robust results. In addition, as a preliminary step to this complete model, we estimated the individual impact of the Territory, Politics and Governance variables on efficiency levels. These results allow us to know the relevance of each group of variables in the efficient management of the pandemic.

**Table 4:** Regression results

	Territory				Politics	Governance	Full model			
*Intercept*	***1.13882252 (39.7714)	***1.20099835 (29.7455)	***1.05303212 (29.1811)	***1.07536113 (23.9262)	***1.18039374 (41.3537)	***1.19211379 (43.4479)	***1.16284408 (38.7763)	***1.22670332 (29.4041)	***1.10365424 (27.4265)	***1.11890372 (23.3499)
Europe	***-0.06346139 (− 2.7537)	***-0.12563723 (− 6.2090)	0.02232901 (1.1001)	–	–	–	*-0.04394037 (− 1.8185)	***-0.10779960 (− 5.1252)	0.01524948 (0.7627)	–
Americas	***-0.08579040 (− 4.7282)	***-0.14796623 (− 6.7889)	–	−0.02232900 (− 1.1001)	–	–	***-0.05918985 (− 2.9290)	***-0.12304908 (− 5.3670)	–	− 0.01524948 (− 0.7627)
Asia	***0.06217583 (2.7625)	–	***0.14796623 (6.7889)	***0.12563723 (6.2090)	–	–	***0.06385923 (2.8670)	–	***0.12304908 (5.3670)	***0.10779960 (5.1252)
Africa	–	***-0.06217583 (− 2.7625)	***0.08579040 (4.7283)	***0.06346139 (2.7537)	–	–	–	***-0.06385924 (− 2.8671)	***0.05918985 (2.9291)	*0.04394037 (1.8186)
Gender	–	–	–	–	0.02873892 (1.2928)	–	*0.03336502 (1.7703)	*0.03336502 (1.7704)	*0.03336502 (1.7704)	*0.03336502 (1.7703)
Ideology	–	–	–	–	−0.01847490 (− 1.3876)	–	−0.00797304 (− 0.7023)	−0.00797304 (− 0.7023)	−0.00797304 (− 0.7023)	−0.00797304 (− 0.7023)
Voice	–	–	–	–	–	***-0.00165806 (−4.8315)	**-0.00087394 (− 2.4875)	**-0.00087394 (− 2.4875)	**-0.00087394 (− 2.4875)	**-0.00087394 (− 2.4875)
Rule of law	–	–	–	–	–	***0.00187573 (4.5877)	**0.00102903 (2.4508)	**0.00102902 (2.4508)	**0.00102903 (2.4508)	**0.00102903 (2.4508)
Median age	***-0.00562925 (− 5.4577)	***-0.00562924 (− 5.4577)	***-0.00562924 (− 5.4577)	***-0.00562925 (− 5.4577)	***-0.00749303 (− 9.9649)	***-0.00861878 (− 8.4901)	***-0.00715986 (− 5.2347)	***-0.00715986 (− 5.2349)	***-0.00715986 (− 5.2349)	***-0.00715986 (− 5.2349)
GDP	*-0.00257486 (−1.7851)	*-0.00257486 (− 1.7852)	*-0.00257486 (− 1.7852)	*-0.00257486 (− 1.7851)	−0.00061309 (− 0.3870)	−0.00139629 (− 0.9318)	*-0.00277236 (− 1.9548)	*-0.00277236 (− 1.9548)	*-0.00277236 (− 1.9548)	*-0.00277236 (− 1.9548)
Tourism	**0.00086839 (2.4614)	**0.00086839 (2.4615)	**0.00086839 (2.4615)	**0.00086839 (2.4614)	*0.00078553 (1.9563)	**0.00094593 (2.4973)	**0.00084939 (2.4560)	**0.00084943 (2.4562)	**0.00084941 (2.4562)	**0.00084941 (2.4562)
Unemployment	***-0.00672044 (− 4.2442)	***-0.00672044 (− 4.2442)	***-0.00672044 (− 4.2442)	***-0.00672044 (− 4.2442)	***-0.00805795 (− 4.4772)	***-0.00718747 (− 4.2797)	***-0.00593488 (− 3.7825)	***-0.00593487 (− 3.7826)	***-0.00593488 (− 3.7826)	***-0.00593488 (− 3.7826)

Significance: ***1%, **5%, *10%, coefficient and (t-value)

With respect to the geographical location of the country, the results of the regression model confirm the above. That is, countries located in *Europe* and the *Americas* show worse results in the efficiency of pandemic management than countries belonging to *Asia* and *Africa*. These results hold for the individual Territory estimate and for the Full model, so that the geographical impact of the country can be considered a relevant factor in the efficient management of the pandemic. There is a small deviation in the results when we estimate the model without the Asia variable, which appears to be less. These results should be interpreted with caution, knowing that the individual management of the pandemic by countries generates differences in the same geographical area.

Another relevant finding has been the effect of female leadership (Full model) on decision making during the pandemic (*Gender*). The qualities of women when facing risky situations, who show temperance and moderation in government actions, may be the cause of a more efficient management. With the available data, during the management of the pandemic we can affirm that countries led by women are more efficient than those led by men. However, there is still a gender gap in government leadership today, with only 10.32% of the countries in our sample being led by women. On the other hand, when faced with situations of these characteristics, governments have no margin for *ideology*, and are forced to implement technical and impartial decisions in favor of the common good. These political factors maintain their significance for both estimates (Politics and Full model).

Countries that are freer in the area of communication, expression or participation (*Voice*) will have citizens well informed and able to argue positions that diverge from those established by the government. Thus, political leaders should work more efficiently and effectively to gain the support of their citizens. However, the results indicate lower efficiency in pandemic management for countries with more *Voice*. In contrast, countries that are more respectful of their *Rule of law* achieve better results in the efficient management of the pandemic. Compliance with established regulations (home confinement, mobility restrictions, space capacity, among others) to curb the spread of the virus allows for the optimisation of available resources.

Focusing now on the control variables (demographics and economics), they maintain their significance and sign in all estimations, with the exception of the *GDP* variable, which loses its significance in the individual *Politics* and *Governance* estimation.

The virulence of the pandemic has been especially intense among the elderly (*Median population*) due to possible previous pathologies or a more deteriorated immune system. Thus, aging countries (older population) have needed more resources to deal with severe cases in this segment of the population, so limiting the response capacity of the health system. Consequently, an increase in the *Median age* implies a lower efficiency in the management of the pandemic, demonstrating that countries with an aging population have not been able to adapt their decisions to the peculiarities of their populations.

On the other hand, the intensity and speed with which events occurred during the first months of the pandemic, and which froze a large part of the productive activity for fear of health collapse, has caused an unprecedented shock to the economy. To this must be added the uncertainty generated in families, companies and governments by the lack of knowledge of the virus - a counterproductive factor for the economy. We might expect those economies that are stronger and more solvent, i.e., those that are better prepared to face contingencies of this magnitude, to manage the pandemic better. Indeed, the results confirm this point, with countries with more vulnerable economies (higher *GDP* variation) reducing efficiency levels.

Countries that receive more revenue from tourism activities (*Tourism*) improve their efficiency. We understand that these countries strive to offer a good image that is capable of continuing to attract a high number of visitors, so proper management of the pandemic will improve their prestige and maintain the economic activity associated with this sector.

The labor market situation (*Unemployment*) is a good example of how families and households are financially able to withstand crisis situations. In this sense, high unemployment rates reduce people’s autonomy, making them more vulnerable and dependent. Thus, countries with an unstable and poorly diversified labor market (higher unemployment rate) are less able to cope with the effects of the pandemic, and so efficiency deteriorates. This may condition management by governments, because not only the victims of Covid-19 should be attended to, but also those affected by the economic situation, which means diversifying efforts and reducing efficiency.

## Conclusions and policy implications

Improving health management is a current priority for governments in view of the situation generated by Covid-19, which affects both people’s health and the economy. The policies implemented to solve this pandemic are many and varied, depending on the governments of each country, and although facing the same problem, the responses have been different. Taking into account the evolution of the pandemic 11 months later, we thought it necessary to evaluate the health management carried out so far in order to better respond to the foreseeable waves of contagion and their effects.

To this end, we calculated the levels of efficiency in the management of health resources and estimated the impact of Territory, Politics and Governance characteristics controlled by demographic and economic variables of the country. We use DEA as the most appropriate technique to obtain efficiency with undesirable outputs (*Cases confirmed* and *Death rate*). We found that the countries that use more resources in the health system obtain worse results in the management of the pandemic. In particular, European and American countries are less efficient than Asian and African countries.

Thus, we confirm that the geographical situation of the country (Territory) as relevant factor for efficient management of the pandemic. With respect to Politics, female leadership of the government seems to incorporate aspects in management that improve efficiency. The Governance represent a fundamental element in solving the health crisis, greater freedom of expression, communication and citizen participation, as well as poor compliance with the rule of law, will hinder the efficient management of the pandemic. On the other hand, we can state that aging populations, vulnerable economies and an unstable labour market before the pandemic reduce efficiency, while countries that obtain more revenues from international tourism will strive to show a solvent image, thus improving their efficiency. In contrast, the ideology of the government leader do not have a significant effect.

These results provide an opportunity for political leaders to reflect on their management during these months of the pandemic in order to identify mistakes and improve the implementation of effective measures. It has been shown that using more resources does not mean managing better; therefore, policymakers need to pay special attention to the use of resources, taking into account the budgetary constraints of the public sector. Moreover, a very important aspect of efficiency is to learn from the past and not to make the same mistakes. Thus, experience in the governance of pandemics is a key factor, with those countries that have managed similar circumstances in the past being the ones that have obtained the best economic and social results.

Finally, the limitations of the research are centered on the volatility of the pandemic and the inadequate information due to methodological differences in data collection. In the coming months and years, when more and better information becomes available, it will be possible to analyse the management of the pandemic in a broader perspective and to identify the causes and effects in each country. Until then, the results of this research offer an opportunity for policy makers to reflect on their management and to try to improve many aspects before it is too late.

## Data Availability

Not applicable.
